# Virulent infection of outbred Hartley guinea pigs with recombinant Pichinde virus as a surrogate small animal model for human Lassa fever

**DOI:** 10.1080/21505594.2020.1809328

**Published:** 2020-08-30

**Authors:** Shuiyun Lan, Wun-Ju Shieh, Qinfeng Huang, Sherif R. Zaki, Yuying Liang, Hinh Ly

**Affiliations:** a Department of Pathology and Laboratory Medicine, Emory University, Atlanta, GA, USA; b Infectious Disease Pathology Branch, Centers for Disease Control and Prevention, Atlanta, GA, USA; c Department of Veterinary and Biomedical Sciences, College of Veterinary Medicine, University of Minnesota, St Paul, MN, USA

**Keywords:** Arenavirus, mammarenavirus, Lassa virus, Pichinde virus, virulence, pathogenesis, pathology, animal model, surrogate model

## Abstract

Arenaviruses, such as Lassa virus (LASV), can cause severe and fatal hemorrhagic fevers (e.g., Lassa fever, LF) in humans with no vaccines or therapeutics. Research on arenavirus-induced hemorrhagic fevers (AHFs) has been hampered by the highly virulent nature of these viral pathogens, which require high biocontainment laboratory, and the lack of an immune-competent small animal model that can recapitulate AHF disease and pathological features. Guinea pig infected with Pichinde virus (PICV), an arenavirus that does not cause disease in humans, has been established as a convenient surrogate animal model for AHFs as it can be handled in a conventional laboratory. The PICV strain P18, derived from sequential passaging of the virus 18 times in strain 13 inbred guinea pigs, causes severe febrile illness in guinea pigs that is reminiscent of lethal LF in humans. As inbred guinea pigs are not readily available and are difficult to maintain, outbred Hartley guinea pigs have been used but they show a high degree of disease heterogeneity upon virulent P18 PICV infection. Here, we describe an improved outbred guinea-pig infection model using recombinant rP18 PICV generated by reverse genetics technique followed by plaque purification, which consistently shows >90% mortality and virulent infection. Comprehensive virological, histopathological, and immunohistochemical analyses of the rP18-virus infected animals show similar features of human LASV infection. Our data demonstrate that this improved animal model can serve as a safe, affordable, and convenient surrogate small animal model for studying human LF pathogenesis and for evaluating efficacy of preventative or therapeutic approaches.

## Introduction

Lassa fever (LF) is an acute viral illness that is endemic in West Africa, where there are approximately 500,000 cases and 5,000 deaths each year [1]. The case-fatality rate is up to 20% among hospitalized patients and can be as high as 50% in sporadic epidemics. LF disease is caused by infection by Lassa virus (LASV), a zoonotic RNA virus within the *Mammarenavirus* genus of the *Arenaviridae* family. The natural reservoir for LASV is the rodent *Mastomys natalensis* [1] or closely related species. Humans can be infected through direct contact with, ingestion or inhalation of contaminated excretia. Several other mammarenaviruses, i.e., Lujo in Africa, Junin, Machupo, Guanarito, Sabia, and Chapare in South America, can cause similar hemorrhagic fever (HF) diseases with high mortality [2]. Currently, there are no effective vaccines, except for Candid#1 against Junin virus used only in Argentina, and limited treatment options for these arenavirus hemorrhagic fevers (AHFs) [3].

AHF diseases have similar clinical courses [4,5]. After an average 10-day incubation period, patients experience a gradual onset of nonspecific syndromes such as fever, general weakness, and headache. A few days later, nausea, vomiting, renal and hepatic disorders, and hemorrhage are often observed. In severe cases, multisystem failure with hypotension and shock can lead to death. Hemorrhage (bleeding) occurs in less than 20% of human Lassa fever cases and normally from mucosal membrane such as conjunctiva, gastrointestine, mouth, nose, and female genital tract. Characteristic skin rashes occur in some patients. The disease can culminate in shock, multisystem organ failure, and death at the terminal stage for some AHF patients.

The pathogenesis of LF disease has not been fully understood. However, several distinct features have been noted. For example, viremia level is closely associated with disease outcome and can accurately predict lethality [6]. The most frequently observed pathological lesions are patchy hepatocellular, splenic, and adrenal necrosis. However, these pathological lesions are generally not severe enough to explain the cause of death [7]. In addition, a general immune suppression in lethal LASV infection is evident from the findings of marked lymphoid cell depletion and necrosis, and the lack of an effective immune response [8].

Several obstacles can impede research on highly pathogenic arenaviruses, which are classified in the Category A Pathogen List and can only be handled in Biosafety Level-4 (BSL-4) facilities. The lack of a convenient immune-competent small animal model has also hampered advancement in understanding AHF pathogenesis. Non-human primate (NHP) is the gold standard animal model for arenaviruses but is limited by high costs and small sample size [8,9]. Laboratory mice generally do not fall sick after infection with pathogenic arenaviruses, except for certain mice with either defective or manipulated immune systems [10]. The 129 Sv mice, which genetically lack interferon α and β receptors (IFNAR^−/-^), can develop a non-lethal acute infection with persistent viremia but not febrile or neurological symptoms upon LASV infection [11–14]. Other mouse strains such as STAT1-/- and IFNAR-/- mice, though succumb to infection, do not develop the HF disease symptoms [13–15]. IFNAR^−/-^ mice that are irradiated and transplanted with bone marrow progenitor cells of the wild type C57BL/6 mice can uniformly succumb to LASV infection [11,16]. A humanized HHD mouse model, which are wild-type C57BL/6 mice expressing a human/mouse chimeric HLA-A2.1, can show some clinical signs similar to human LF upon LASV infection [17]. Additionally, the CBA mice, upon intracerebral infection with LASV, can produce 80–100% lethality at 7 to 9 days post infection [18], but the disease signs (body weight loss and paralysis) are more consistent with the central neurological diseases caused by another arenavirus pathogen lymphocytic choriomeningitis virus (LCMV) than the multi-systemic disease of human LF.

Indeed, several laboratories have used LCMV to develop surrogate animal models for LF (reviewed in [19]). For example, the LCMV (WE strain) can cause a disease in rhesus macaques [20] and HHD mice [21] that reproduces certain aspects of LASV pathogenesis such as vascular leakage. However, the requirement of a BSL-3 biosecurity and biosafety to work with LCMV (WE strain) limits its use. A less pathogenic LCMV strain (Clone 13) (BSL-2) has been reported to reproduce some aspects of LF pathology, such as thrombocytopenia, hepatocellular and splenic necrosis, and cutaneous hemorrhage in FVB/N mice [22], thrombocytopenia and vascular leakage in NZB mice [23], as well as vascular leakage and respiratory failure in PL/J, SL/J, and CC NZO mice [24]. The disease pathogenesis in these LCMV-infected mice appears to be immunopathology, as death can be aborted by blockade of interferon-1 (IFN-1) signaling or by deletion of CD8 T cells [24], which is different from the generalized immune suppression seen in severe and lethal human LF infection.

Immunologically competent inbred Strain 13 guinea pigs have been used as an alternative small rodent model for LF [25–27], but they are not readily available and lack the genetic diversity mimicking humans. Outbred Hartley guinea pigs are commercially available and have diverse genetic background. LASV-infected Hartley guinea pig model shows uniform lethality and clinical symptoms, such as fever, followed by hypothermia just before death, as well as weight loss, lethargy, thrombocytopenia, neutropenia, and lymphopenia [28], but these studies are relatively expensive and require high biocontainment facilities.

Jahrling and colleagues originally developed a safe, convenient, and economical surrogate animal model for LF, which is based on infection of inbred strain 13 guinea pigs with Pichinde virus (PICV) [29,30], a nonpathogenic BSL-2 arenavirus that is not known to cause disease in humans [31]. Sequential passages of PICV in the spleen of inbred strain 13 guinea pigs led to a variant strain (i.e. P18, passaged 18 strain), which caused severe disease in guinea pigs [29] that mimics human LF infection [32] in many aspects, such as fever, leukopenia, thrombocytopenia, and a general immune suppression [33–38]. Due to the high cost and limited availability of strain 13 inbred guinea pigs, Aronson and colleagues demonstrated that the virulent P18 strain of PICV could cause a similarly virulent infection of outbred Hartley guinea pigs [39]. However, while strain 13 guinea pigs showed 100% mortality upon P18 virus infection, outbred Hartley guinea pigs had an average mortality rate of 50% to 70% [40,41], which could limit the accurate analysis of the early host responses to virulent viral infection. Such variation is likely due to the genetic heterogeneity among outbred animals.

We have previously developed infectious clones for both avirulent P2 (passaged 2) strain of PICV and the virulent P18 PICV strain and demonstrated that these recombinant rP2 and rP18 viruses reproduced the differential disease outcomes of the parental P2 and P18 strains in outbred Hartley guinea pigs [42]. By taking advantage of the convenience of the reverse genetics technology, we have generated different recombinant virus variants to help identify the molecular determinants of rP18 virulence in guinea pigs [43–45]. In the present study, we show that the recombinant rP18 virus, generated by reverse genetics technique followed by plaque purification, can produce a consistent disease outcome in outbred Hartley guinea pigs (~95% mortality), as well as disease phenotypes and pathologies that closely mimic fatal human Lassa fever cases. Thus, this refined rP18-virus infected outbred guinea pig model can serve as a valuable tool to conveniently determine the biological roles of viral and host factors in arenavirus virulence and LF disease pathogenesis.

## Materials and methods

### Viruses and cells

Baby hamster kidney cells BHK21 and African green monkey kidney Vero cells were maintained in DMEM media supplemented with 10% FBS and 50 μg/ml penicillin/streptomycin. BSRT7-5 cells, which are BHK-21 cells engineered to stably express the T7 RNA polymerase, were obtained from K.K. Conzelmann (Ludwig-Maximilians-Universität, Germany) and cultured in minimal essential media (MEM) (Invitrogen-LifeTechnologies) supplemented with 10% fetal bovine serum (FBS), 1 μg/ml Geneticin (Invitrogen-LifeTechnologies), and 50 ug/ml penicillin-streptomycin. At every second passage, 1 mg of G418 per ml of cell media was added to the cells.

Recombinant rP2 and rP18 viruses were generated from the respective virus infectious clones via reverse genetics technique as described previously [42]. Briefly, BSRT7 cells were transfected in 6-well plates via Lipofectamine 2000 reagent (Invitrogen) with two plasmids encoding the antigenomic strands of the PICV large (L) and small (S) segments that were transcribed by the T7 RNA polymerase. Cell media containing the viral supernatants collected at 48 h and 72 h after transfection were used to conduct plaque assay on Vero cells as described below. Single plaques were selected for amplification by directly infecting BHK-21 cells in 10-cm plates for 48 h. The cell media containing the virus supernatants were harvested, filtered through a 0.2-um filter, and stored at −80°C as virus stocks used for animal infection. Only virus stocks stored for less than 3 months were used for animal studies in order to minimize potential attenuation phenotype of the aged virus samples.

### PICV plaque assay

Vero cells were seeded into six-well plates at 90% to 100% confluency and infected with 0.5 ml of 10-fold serial dilutions of viruses in MEM complete media for 1 h at 37°C. After removing the media, the cells were incubated in fresh media supplemented with 0.5% agar and cultured for 4 days at 37°C. Plaques were stained overnight with diluted neutral red solution (1:50) in 0.5% agar–MEM–10% FBS.

### In vivo experiment

All animal experiments were conducted following the guidelines and approved protocols from the Institutional Animal Care and Use Committee (IACUC) at Emory University and University of Minnesota, Twin Cities. Healthy 300–350 gram outbred Hartley guinea pigs purchased from Charles River Laboratories were infected intraperitoneally (IP) with 10,000 PFU of avirulent rP2 or virulent rP18 PICVs. Rectal temperature and body weight were measured daily until day 18 post infection (18 dpi). As relatively young guinea pigs were used, the body weight of infected animals could be monitored and compared to that of the mock-infected animals (by IP injection with phosphate buffer saline or PBS) that were growing at an expected rate and therefore could be used as a nomogram for a comparison. Guinea pigs were declared moribund and euthanized if body weight decreased by 30% compared to the nomogram. Blood were collected for assessment of a complete blood count (CBC) by Department of Pathology, Emory University, and of a coagulation test by Antech Diagnostics (Irvine, CA). Necropsy was performed on moribund or control animals, in which organs were removed for evaluation of gross pathology and tissues were collected for viral and histopathological analyses.

### Histopathological analysis

- Human tissue samples available in the CDC archive that included four autopsy cases from 1996–1997 Sierra Leone Lassa Fever outbreak, collected with consents from families of the patients, were used in the current study. Some of these archived and de-identified tissue samples were fixed in 4% formaldehyde, embedded in paraffin, sectioned, stained with hematoxylin and eosin (H&E), and examined microscopically for histopathological changes. For immunohistochemical (IHC) analysis of viral antigens, paraffin-embedded tissue sections or cells were subjected to antigen retrieval followed by staining with mouse anti-PICV serum or a mouse monoclonal antibody targeting LASV glycoprotein 2 (CDC). Tissue sections were sequentially treated with biotinylated secondary antibodies, alkaline phosphatase-conjugated streptavidin, and napthol fast red according to the manufacturer’s protocol (LSAB2 Universal Alkaline Phosphatase kit, DAKO, Carpinteria, CA), and counterstained in Meyer’s hematoxylin.

### Statistical analyses

Statistical analysis of the survival curve by log-rank (Mantel-Cox) χ2 test was conducted using GraphPad Prism 5 software. Statistical significance between two groups was determined using the Student’s t-test.

## Results & discussion

### Clinical symptoms and mortality of recombinant Pichinde (rP2 or rP18) virus infections of outbred Hartley guinea pigs

**Figure 1.**
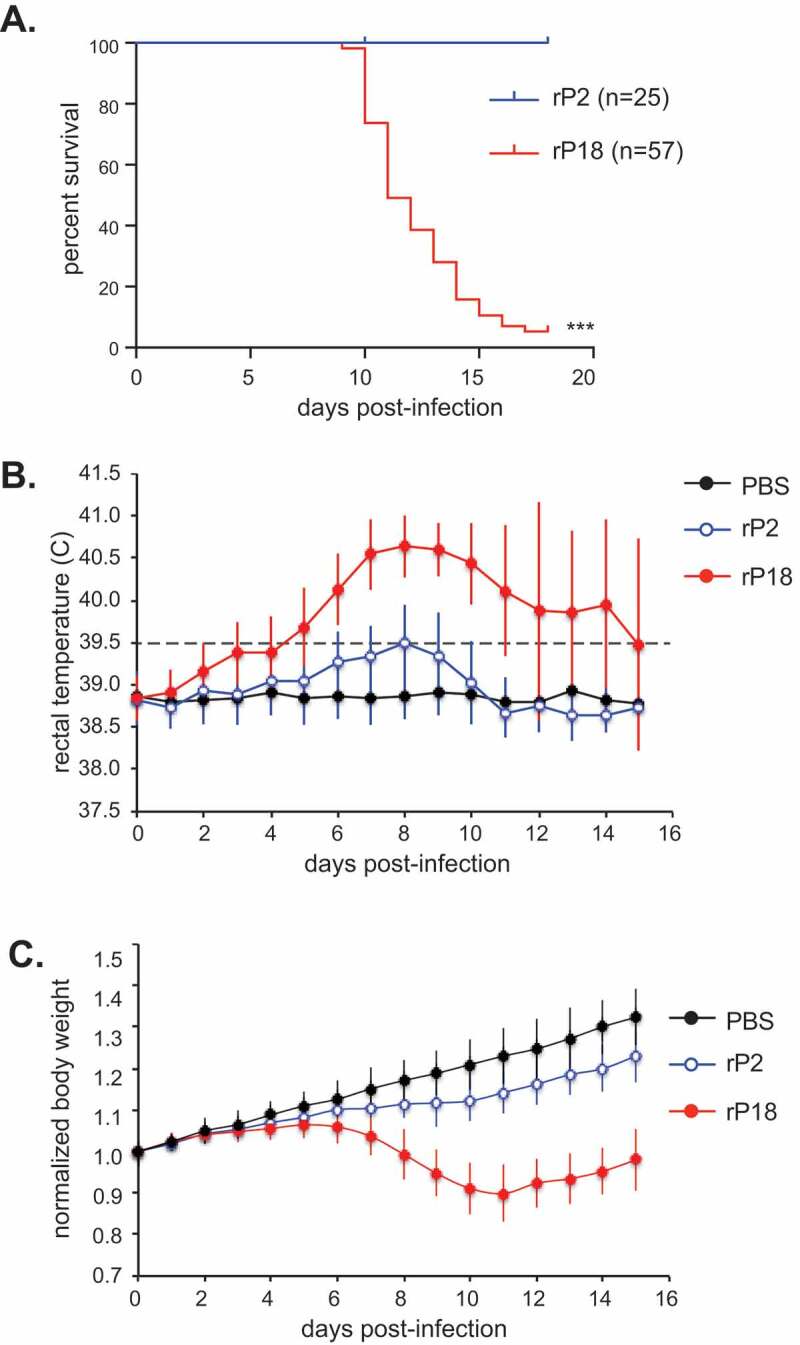
Mortality and clinical symptoms of recombinant Pichinde virus (rP2 and rP18) infections in outbred Hartley guinea pigs. Guinea pigs were mock infected (PBS) or infected via IP route with 10,000 pfu of rP2 or rP18. (a) Survival curve plotted for rP2 (n = 25) and rP18 (n = 57). (b) Rectal temperature (°C) monitored during the experiment. Temperature > 39.5°C was considered feverish and identified by a dashed line. (c) Body weight monitored during the experiment. Body weight of each animal was normalized to its body weight at day 0 (set as 1.0). Data shown were the average of animals in replicate studies using fresh batches of recombinant viruses that were conducted between 2008 and 2015, with error bars representing standard deviation.

Recombinant rP2 and rP18 PICVs generated via reverse genetics technique were plaque purified [42]. Healthy outbred Hartley guinea pigs intraperitoneally (IP) were infected with 10,000 PFU of recombinant rP2 or rP18 PICV. Data described in this manuscript are those from studies conducted between 2008 and 2015 using fresh batches (less than 3 months old) of recombinant rP2 and rP18 PICVs. Cumulatively, all rP2-virus infected animals (n = 25) survived the infection, whereas 54 out of 57 rP18-virus infected animals reached terminal points during the 18-day cycle of infection and were euthanized (Figure 1a). The approximately 95% mortality rate observed in this rP18-virus infected guinea pig model was significantly higher than the average 50%-70% mortality rate reported for the P18 spleen-isolated virus stock to infect the outbred Hartley guinea pigs via the same (IP) route [40]. The rP18-vius infected animals reached terminal points as early as 10 days post infection (dpi), with a median survival of 11 days (Figure 1a). rP18 virus infection led to early onset (as early as 3 dpi) and prolonged fever (average 9 dpi), while rP2 caused only a brief febrile reaction (Figure 1b). As relatively young guinea pigs were used, we were able to monitor body weight of infected animals and compared to that of the PBS mock-infected animals that were growing at an expected rate. The rP2-virus infected guinea pigs continued to gain weight, though at a slightly lower rate than those in the PBS mock-infected control group (Figure 1c). The rP18-virus infected guinea pigs had a similar grow rate as rP2-virus infected animals during the first 5 days post-infection but they started to show body weight loss at day 6 and subsequent (progressive) body weight loss to 30% of animals in the PBS control group (Figure 1c), which was deemed appropriate for euthanization. Taken together, these results suggest that tissue culture generated and plaque-purified recombinant PICVs can cause highly consistent disease outcomes and mortality in outbred Hartley guinea pigs.

### Gross pathology

**Figure 2.**
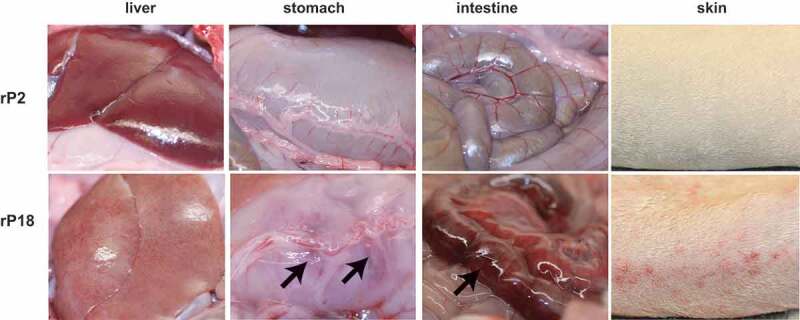
Gross Pathology of rP2 and rP18-infected guinea pigs.

Necropsy of rP2- and rP18-virus infected guinea pigs revealed gross pathology mainly in the liver. Livers from the moribund rP18 virus-infected animals were pale with red speckles as compared to the normal uniformly dark red color of those from animals infected with the avirulent rP2 PICV (Figure 2, left panel). This appears to be consistent with the major and most common pathologic changes in the liver of patients fatally infected with Lassa fever [7,46]. Whereas previously established outbred PICV-guinea pig model did not show consistent hepatic damage in the moribund animals [30], our outbred guinea pigs infected with rP18 PICV showed consistent and marked gross morphological changes in the liver. We also consistently observed evidence of internal hemorrhages from organs such as stomach and intestine (Figure 2, shown with arrows) and occasional skin petechiae in a relatively small number of the rP18-virus infected animals (Figure 2, right). These are specific features reminiscent of LF in humans but have not been reported in any of the previously established inbred or outbred guinea pig infection models [29,30].

For rP2 infection, images of liver, stomach, intestine, and skin are from two rP2-infected animals, which stayed healthy and were euthanized at day 14 pi. For rP18 infection, the images were from animals euthanized after reaching terminal points. The liver was from a guinea pig euthanized at day 11 pi; the stomach was from a guinea pig euthanized at day 12 pi; the large intestine was from a guinea pig euthanized at day 13 pi; the skin was from a guinea pig euthanized at day 14 pi.

### Histopathology

**Figure 3.**
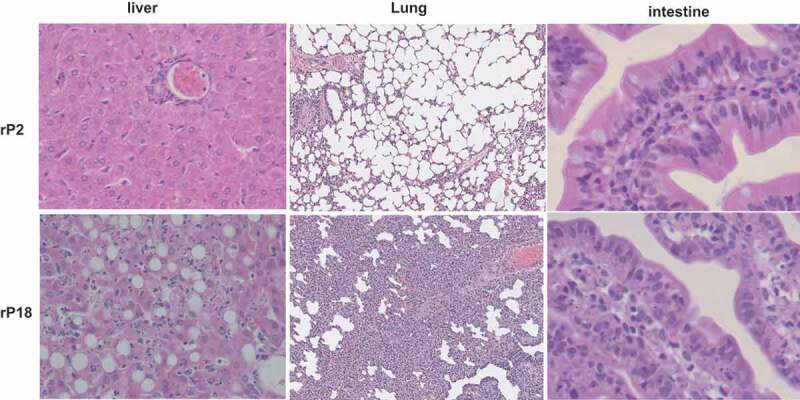
H&E staining of liver, lung, and intestine.

Histopathological characterization of livers of moribund animals of the virulent rP18 virus infection demonstrated massive liver injury and necrosis that were characterized by extensive fatty changes, hepatocellular necrosis, and also evidence of apoptosis and scattered lymphocyte infiltration (Figure 3, left panel), similar to the reported cases of acute liver injury seen in severe and lethal forms of LF [46,47]. In addition to the liver, other major organs also exhibited similar histopathological changes as observed in lethal human LF. For example, the lung showed a mild to moderate increase of interstitial and peribronchiolar inflammatory infiltrates (Figure 3, middle panel). The gastrointestinal tract showed focal submucosal edema, increased inflammatory infiltrates in the lamina propria, hyperplastic mesothelial cells on serosal surface, and apoptotic cells suggesting epithelium damage (Figure 3, right panel). In contrast, rP2-virus infected outbred Hartley guinea pigs did not show any significant histopathological changes in any of the tissues analyzed.

These images were from one rP2-infected guinea pig that stayed healthy and was euthanized at Day 18 pi, and from one rP18-infected guinea pig that was euthanized at Day 15 pi after reaching terminal points.

### Thrombocytopenia and lymphopenia of rP18-virus infected outbred guinea pigs

We conducted complete blood count (CBC) analysis of the blood samples collected from rP2- and rP18-virus infected outbred Hartley guinea pigs at day 15 post-infection, when the rP18-virus infected animals became moribund. Shown in Table 1, rP18-virus infected animals had normal numbers of red blood cells (RBCs), but significantly decreased numbers of platelets (PLT) and white blood cells (WBCs), at ~25% level of rP2-virus infected animals. In particular, lymphocytes (LYMF) decreased in both absolute number and percentage in the WBC population. The laboratory findings of thrombocytopenia and lymphopenia in rP18-virus infected outbred guinea pigs were similar to those in AHF patients including those with Lassa fever [32,48].

**Table 1.** Complete blood count (CBC) of recombinant Pichinde (rP2 or rP18) virus-infected outbred Hartley guinea pigs*.

Virus strains	RBC 10^6^/mm^3^	PLT 10^3^/mm^3^	WBC 10^3^/mm^3^	LYMF 10^3^/mm^3^	LYMF %	GRAN 10^3^/mm^3^	GRAN %	MONO 10^3^/mm^3^	MONO %
rP18	3.9 ± 0.9	76.5 ± 33.7	3.0 ± 1.8	0.7 ± 0.7	24.5 ± 8.1	1.8 ± 0.9	61.7 ± 8.4	0.5 ± 0.3	13.9 ± 0.7
rP2	4.6 ± 0.8	318 ± 94.4	11.2 ± 4.0	5.1 ± 1.9	46.7 ± 12.4	4.6 ± 2.2	40.9 ± 9.0	1.4 ± 0.7	12.4 ± 4.0

* Blood were collected at the terminal points of rP18-virus infected animals and from rP2-virus infected animals on the same day. Data shown were the average from at least 3 independent experiments. RBC, red blood cells; PLT, platelets; WBC, white blood cells; LYMF, lymphocytes; GRAN, granulocytes; MONO, monocytes. Lymphocytes (LYMF), granulocytes (GRAN), and monocytes (MONO) are shown in number (10^3^/mm^3^) and in percentage of white blood cells (WBCs).

### Coagulopathy in rP18-virus infected outbred guinea pigs

We observed significant coagulation defects in rP18-virus infected outbred Hartley guinea pigs. In the extreme cases, blood failed to clot in the absence of heparin when it was collected. We collected blood from two moribund animals after virulent rP18-virus infection and one control animal that recovered from avirulent rP2-virus infection. A coagulation test conducted by Antech Diagnostics showed that, when compared to the rP2-virus infected healthy guinea pig, both moribund rP18-virus infected animals had prolonged partial thromboplastin time (PTT), prolonged prothrombin time (PT), decreased fibrinogen level, but seemingly normal D-dimer level (Table 2). Our findings are consistent with a previous study with P18-virus infected outbred guinea pigs [33] in demonstrating the significant coagulation abnormalities associated with LF.

**Table 2.** Coagulation assays of blood collected from recombinant (rP2 or rP18) virus-infected outbred Hartley guinea pigs*.

Animal ID	Virus	Morbidity	PTT (sec)	PT (sec)	Fibrinogen (mg/dl)	D-dimer (ng/ml)
855956	rP2	Healthy	11.4	31.2	215	<250 (normal)
855959	rP18	Moribund	>100	>50	113	<250 (normal)
855961	rP18	Moribund	>100	>50	113	<250 (normal)

* Blood were collected at day 15 post-infection from two rP18-virus infected guinea pigs and one rP2-virus infected animal and analyzed by Antech Diagnostics.

### Viral replication in guinea pigs

Virulent rP18 PICVs were found at high levels in all organs of moribund guinea pigs examined, ranging from 3.1 × 10^6^ pfu/g in lymph nodes to 3.2 × 10^8^ pfu/g in adrenal gland (Table 3). The highest levels of virus were found in the liver and adrenal gland. In contrast, no virus was detected by plaque assay (detection limit of < 20 PFU/g) in rP2-virus infected animals that were sacrificed at the same time. Similar to postmortem findings on fatal human LF infections in which viruses were pantropic [7], rP18 viruses also infected and spread to most if not all tissues in guinea pigs.

**Table 3.** Viral titers (PFU/g) in tissues of rPICV-infected outbred Hartley guinea pigs*.

	Heart	Lung	Liver	Spleen	Stomach	Pancreas	Adrenal gland	Kidney	Lymph node
rP2	<20	<20	<20	<20	<20	<20	<20	<20	<20
rP18	6.5E+06	6.7E+07	1.9E+08	3.0E+07	5.6E+07	6.5E+06	3.2E+08	3.9E+06	3.1E+06

* The tissues were collected at the terminal points of rP18-virus infected animals and from rP2-virus infected animals at day 15. Virus titers in tissue homogenates were determined by plaque assay and shown as plaque-forming unit (PFU) per gram of tissue. Data shown were the average from at least 3 independent experiments.

**Figure 4.**
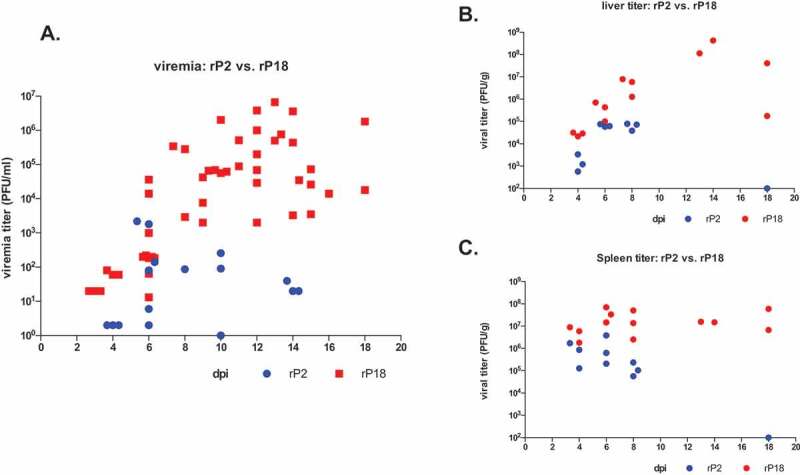
Kinetics of virus replication in rP2- and rP18-infected animals. Sera, livers, and spleens from the rP2- and rP18-infected animals at different days during the course of the experiment or at terminal points were collected for viral titer quantification by plaque assay.

We collected sera, livers, and spleens from the rP2- and rP18-infected animals at different days during the infection or at terminal points and quantified the viral titers by plaque assay (Figure 4). Shown in Figure 4a, viremia could be detected as early as 3 dpi in rP18-infected guinea pigs and the viremia level quickly increased to relatively high levels afterward, with up to 5 × 10^4^ PFU/ml at 6 dpi and between 1 × 10^5^ PFU/ml and 1 × 10^6^ PFU/ml at 10 dpi and the subsequent terminal points. In sharp contrast, the rP2-infected guinea pigs did not show obvious viremia until 6 dpi with up to 2,000 PFU/ml in some animals and did not support further increase of viremia afterward. A very low level of viremia (up to 100 PFU/ml) was occasionally detected at 10 dpi and 14 dpi in some rP2-infected animals and no viremia were detected at the end of the experiment (18 dpi). The high viremia levels were associated with virulent rP18 virus-induced mortality in guinea pigs, which is consistent with clinical reports that high level of viremia is a poor prognosis in LASV-infected patients [6]. The kinetics of rP18 virus growth in the livers (Figure 4b) were similar to the viremia level, in which the virus was detected at 4 dpi and increased rapidly afterward and remained high until the terminal points. The titers of the rP2 virus were also detected in the liver of infected animals at 4 dpi and increased, but to a lower level than rP18-infected animals, from 6 dpi to 8 dpi, and were completely absent at the end of the experiment (18 dpi) (Figure 4b). A slightly different kinetic was observed for viral replication in the spleens (Figure 4c), where rP18 and rP2 already reached high levels (~1x10^7^ PFU/g for rP18 and 1 × 10^6^ PFU/g for rP2) at 3 dpi, which was the earliest sampling time point. Subsequently, the rP18 titer increased slightly and remained high until the terminal points, while the rP2 titer decreased and was completely absent at 18 dpi. That rP18 peaked at 4 dpi – 6 dpi in the spleen and at 10 dpi – 12 dpi in the liver and blood suggests that early viral replication occurs in the spleen and lymphatic system before spreading to other major organs and tissues. Taken together, our results suggest that the virulent rP18 virus replicates uncontrollably in guinea pigs while the rP2 virus shows a limited ability to replicate *in vivo* that can be cleared quickly by the host.

### Immunohistochemical (IHC) analysis of viral antigen distribution in tissues

**Figure 5.**
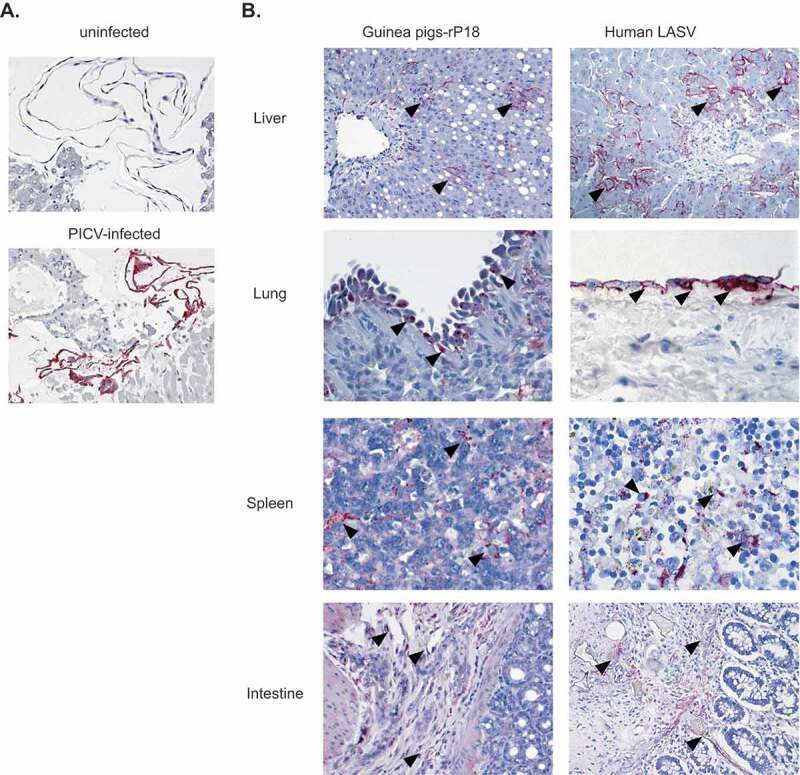
IHC staining of liver, lung, spleen, and intestine from rP18-infected guinea pig and LASV-infected human. (a) Mouse anti-PICV serum was used to validate its specificity to detect PICV antigens via IHC of PICV-infected Vero cells (bottom panel, red color) versus mock (uninfected) Vero cells (top panel). The same mouse anti-PICV serum was used in IHC to detect PICV antigens in guinea pig tissue sections prepared from a representative rP18-infected guinea pig that was euthanized at day 15 pi after reaching terminal points. Immunostaining of PICV antigens is seen in hepatocytes and sinusoidal lining cells in liver; mesothelial cells on pleural surface of lung; dendritic cells in spleen; endothelial cells in submucosa of intestine. The examples of immunolocalization in each photomicrograph are highlighted by arrowheads.

We conducted IHC to determine the distribution of viral antigens in the infected animal tissues. The specificity of the mouse anti-PICV serum used to detect PICV antigens was demonstrated by IHC analysis performed on PICV-infected and mock-infected Vero cells (Figure 5a). The same IHC method was applied to detect PICV antigens in guinea pig tissue sections prepared from a representative rP18-infected guinea pig that was euthanized at day 15 pi after reaching terminal points (Figure 5b). Immunostaining of PICV antigens is seen in hepatocytes and sinusoidal lining cells in liver; mesothelial cells on pleural surface of lung; dendritic cells in spleen; endothelial cells in submucosa of intestine. The examples of immunolocalization in each photomicrograph are highlighted by arrowheads. The distribution of PICV antigen in each of these tissues (Figure 5b, left panels) was similar, if not identical, to that of LASV antigen seen in fatal human LASV infection (Figure 5b, right panels). Our data were consistent with the previous reports with P18 virus infection of inbred guinea pigs and human Lassa virus infections [7,29], and together demonstrated that both PICV and LASV could replicate in all extraneural tissues of the infected hosts and had identical distribution of viral antigens within their infected tissues.

In summary, our current study provides clinical, pathological, immunohistochemical, hematological, and viral data to demonstrate the similarity of the virulent PICV-infected guinea pigs and lethal human LF, highlighting the relevance of this convenient surrogate small animal model in understanding the LF disease pathogenesis. In addition, we have shown that recombinant PICVs generated by reverse genetics technique followed by plaque purification generate consistent disease outcomes when they are used to infect outbred Hartley guinea pigs, a feature that is useful in the analysis of early host immune responses associated with virulent arenavirus infection *in vivo* and for testing of preventative or therapeutic treatments against lethal LF.

While this manuscript was under review, we published another study that focused on extensive histopathological and immunochemical analyses of some representative tissues collected from a small group of guinea pigs (N = 6) that were infected with 6 different viruses, P2, rP2, P18, rP18, or two reassortant viruses (rS2L18 and rS18L2) [49]. On the contrary, the current study reports a comprehensive analysis of a large number of rP2- and rP18-infected guinea pigs (N = 57 for rP18 and N = 25 for rP2) over a period of 7 years to document their clinical symptoms, mortality, pathology, histopathology, hematology, coagulation, viral growth, and more importantly conducts a direct comparative gross- and histopathological and immunochemical analyses of tissues and organs collected from rP2- and rP18-infected animals as well as some archived tissues of lethal human LF cases. Independent data sets and images from different PICV-infected guinea pigs are shown. As such, there are no overlaps in data reporting and the study designs of the two papers are different, yet they both highlight the importance of this convenient surrogate small animal model for studying human LF disease pathogenesis.
